# Addition of platinum derivatives to neoadjuvant single-agent fluoropyrimidine chemoradiotherapy in patients with stage II/III rectal cancer: protocol for a systematic review and meta-analysis (PROSPERO CRD42017073064)

**DOI:** 10.1186/s13643-018-0678-9

**Published:** 2018-01-22

**Authors:** Felix J. Hüttner, Pascal Probst, Eva Kalkum, Matthes Hackbusch, Katrin Jensen, Alexis Ulrich, Markus W. Büchler, Markus K. Diener

**Affiliations:** 1 Department of General, Visceral and Transplantation Surgery, University of Heidelberg, Im Neuenheimer Feld 110, 69120 Heidelberg, Germany; 2 The Study Center of the German Surgical Society (SDGC), University of Heidelberg, Im Neuenheimer Feld 130.3, 69120 Heidelberg, Germany; 3 Institute of Medical Biometry and Informatics (IMBI), University of Heidelberg, Im Neuenheimer Feld 130.3, 69120 Heidelberg, Germany

**Keywords:** Rectal cancer, Neoadjuvant therapy, Chemoradiotherapy, Survival, Treatment toxicity

## Abstract

**Background:**

Neoadjuvant (chemo-)radiation has proven to improve local control compared to surgery alone, but this improvement did not translate into better overall or disease-specific survival. The addition of oxaliplatin to fluoropyrimidine-based neoadjuvant chemoradiotherapy holds the potential of positively affecting survival in this context since it has been proven effective in the palliative and adjuvant setting of colorectal cancer. Thus, the objective of this systematic review is to assess the efficacy, safety, and quality of life resulting from adding a platinum derivative to neoadjuvant single-agent fluoropyrimidine-based chemoradiotherapy in patients with Union for International Cancer Control stage II and III rectal cancer.

**Methods:**

MEDLINE, Web of Science, and Cochrane Central Register of Controlled Trials will be systematically searched to identify all randomized controlled trials comparing single-agent fluoropyrimidine-based chemoradiotherapy to combined neoadjuvant therapy including a platinum derivative. Predefined data on trial design, quality, patient characteristics, and endpoints will be extracted. Quality of included trials will be assessed according to the Cochrane Risk of Bias Tool, and the GRADE recommendations will be applied to judge the quality of the resulting evidence. The main outcome parameter will be survival, but also treatment toxicity, perioperative morbidity, and quality of life will be assessed.

**Discussion:**

The findings of this systematic review and meta-analysis will provide novel insights into the efficacy and safety of combined neoadjuvant chemoradiotherapy including a platinum derivative and may form a basis for future clinical decision-making, guideline evaluation, and research prioritization.

**Systematic review registration:**

PROSPERO CRD42017073064

**Electronic supplementary material:**

The online version of this article (10.1186/s13643-018-0678-9) contains supplementary material, which is available to authorized users.

## Background

Colorectal cancer is one of the most common malignant diseases in industrialized nations, with an estimated number of > 135,000 new cases and > 50,000 deaths in the USA for the year 2017. Thus, it is one of the three most common causes of cancer deaths in adults of both sexes [1, 2]. Furthermore, colorectal cancer has a tremendous economic impact accounting for approximately € 6 billion costs due to lost productivity in Europe 2008, because of cancer-related premature mortality [3].

About one third of all colorectal carcinomas are located in the rectum and therefore require specific treatment algorithms [1, 2]. The routine use of total mesorectal excision has served to increase survival and reduce the rates of local recurrence in this patient population [4, 5]. Furthermore, international and national guidelines on colorectal cancer recommend neoadjuvant therapy consisting of either short-term radiation (5 × 5 Gy) or combined chemoradiotherapy (usually with a total of 50.4 Gy together with fluoropyrimidine-based chemotherapy) for the treatment of all patients with Union for International Cancer Control (UICC) stage II or III rectal cancer [6, 7]. Multimodal treatment strategies including neoadjuvant chemoradiotherapy have contributed to reduce rates of local recurrence in cases of nodal-positive disease or an advanced T stage. However, neither neoadjuvant radiation nor neoadjuvant chemoradiotherapy was able to improve overall survival, which is the ultimate goal of any cancer-directed therapy [8–10]. This led to different approaches in the recent use of neoadjuvant chemoradiotherapy. While some clinicians favored a more selective indication for neoadjuvant chemoradiotherapy, e.g., based on preoperative radiologic assessment of the circumferential resection margin [11–13], others started to exert and investigate more effective chemotherapeutic agents in the setting of preoperative chemoradiotherapy [14, 15]. This second approach aims at reducing rates of not only local recurrence but also distant recurrence, which remain at a high level of up to 30% after curative resection of advanced rectal cancer.

Oxaliplatin is one of these chemotherapeutic agents, which has proven to be effective in the palliative and adjuvant setting of colorectal cancer by improving progression-free or disease-free survival, respectively [16, 17]. Thus, the addition of oxaliplatin to fluoropyrimidine-based neoadjuvant chemoradiotherapy holds the potential of positively affecting survival in the neoadjuvant context [18]. However, especially in the light of rising numbers of long-term rectal cancer survivors, the impact of such intensified chemotherapeutic regimens on quality of life and long-term toxicities have to be taken into account in the evaluation of its benefits. Several phase I trials have shown feasibility and acceptable toxicity of this chemotherapeutic regimen [19, 20]. Furthermore, short-term surrogate outcome parameters such as pathological response rate were improved by the combination of oxaliplatin and fluoropyrimidine in phase II trials [21–23]. Therefore, several large, multicenter phase III trials addressed clinical efficacy of this therapeutic concept during the last decade [15, 24, 25] and long-term clinical results have been recently published [14, 26, 27].

One previous systematic review on this topic has evaluated short-term results of four clinical trials evaluating neoadjuvant chemoradiotherapy with fluoropyrimidine alone or in combination with oxaliplatin in locally advanced rectal cancer [28]. The primary outcome evaluated was pathologic complete response, which may be considered a surrogate efficacy endpoint only, and previous research has shown that it does not reliably predict long-term outcome [29]. Furthermore, a recent Cochrane review also only evaluated short-term results of the same four trials and qualitatively analyzed the long-term results of one of the trials [30]. With the recent publication of 3- to 5-year overall and disease-free survival outcomes for several trials [14, 26, 27], a well-conducted systematic review and meta-analysis of the evidence, with survival as primary outcome, has the potential to change current treatment paradigms in Germany and internationally.

Thus, the main objective of this systematic review is to assess the efficacy, safety, and quality of life resulting from adding a platinum derivative to neoadjuvant single-agent fluoropyrimidine-based chemoradiotherapy in patients with UICC stage II and III rectal cancer.

## Methods/design

The review protocol has been registered prospectively in PROSPERO (Registration number PROSPERO 2017: CRD42017073064) and was prepared according to the PRISMA-P statement [31], with a checklist included as Additional file 1. This systematic review and meta-analysis is funded by the German Federal Ministry of Education and Research (www.bmbf.de, project number: 01KG1710).

### Systematic literature search

A systematic literature search will be performed using the validated methods of the Cochrane Collaboration [32]. All stages of study selection, quality assessment, and data extraction will independently be carried out by two reviewers to minimize errors. Any disagreement will be resolved by consensus or by consulting a third reviewer.

The following electronic bibliographic databases will be searched: MEDLINE, Web of Science, and Cochrane Central Register of Controlled Trials (CENTRAL). Reference lists of relevant articles and related systematic reviews will be hand-searched. Clinical trial registries (www.clinicaltrials.gov, www.clinicaltrialsregister.eu, etc.) will be searched for ongoing or unpublished trials.

The PICO (participants, interventions, comparisons, and outcomes) model was used to formulate the research question [32]. Table 1 shows the PICO question together with the search strategy for MEDLINE (via PubMed), which was built using a combination of index or free text words and MeSH terms. The search strategies for other databases were constructed correspondingly. No language or other restrictions including date of publication will be applied.

**Table 1:** PICO question and search strategy for MEDLINE (via PubMed)

P	Patients with stage II/III rectal cancer
I	Neoadjuvant chemoradiotherapy with a platinum derivative added to fluoropyrimidine
C	Neoadjuvant chemoradiotherapy with a single-agent fluoropyrimidine
O	Overall survival, disease-free survival
(neoadjuvant [tw] OR neo-adjuvant [tw] OR preoperative [tw] OR pre-operative [tw] OR “Neoadjuvant Therapy” [MeSH]) AND carboplatin* [tw] OR oxaliplatin* [tw] OR cisplatin* [tw] OR platin* [tw] OR “Carboplatin” [MeSH] OR “Cisplatin” [MeSH] OR “Organoplatinum Compounds” [MeSH] AND ((adenocarcinom* [tw] OR carcinom* [tw] OR cancer [tw] OR malignant growth [tw] OR tumor [tw] OR tumour [tw]) AND (rectum [tw] OR rectal [tw] OR rectum [MeSH])) OR rectal neoplasms [MeSH] AND random* [tw] OR randomized controlled trial [pt] OR RCT [tw] OR “Randomized Controlled Trials as Topic” [MeSH] OR controlled clinical trial [pt] OR clinical trials as topic [mesh: noexp] OR controlled trial [tw] OR clinical trial [tw] OR controlled study [tw] OR control group [tw] OR “Control Groups” [MeSH])	

Following eligibility assessment of the combined search results from all databases, the eligible articles will be complemented searching the Science Citation Index (via Web of Science) for publications that cite trials eligible for inclusion.

We expect to include between four and ten eligible randomized controlled trials (RCTs), with several publications describing results for each trial. A minimum number of four RCTs have been identified by the preceding systematic review by An et al. [28], and at least one additional trial has been identified by our preliminary literature search [26] (Table 2).

**Table 2:** Trials already known about at the start of review

Trial acronym	First author	Country	Trial period
STAR-01	Aschele C. [24]	Italy	11/2003–08/2008
ACCORD 12/0405 PRODIGE 2	Gerard J. P. [14, 25]	France	11/2005–07/2008
NSABP R-04	Allegra C. [44]	USA	07/2004–08/2010
CAO/ARO/AIO-04	Rödel C. [15, 27]	Germany	07/2006–02/2010
n.a.	Jiao D. [26]	China	07/2007–07/2010

### Study selection

Two reviewers will screen all titles and abstracts that have been retrieved by the systematic literature search independently. Retrieved references will be stored in a file of the reference management software EndNote™. Duplicates will be removed, and all other references will be put into specific folders (*inclusion*, *exclusion with reason*, etc.) after screening. The full-text manuscript of all trials that are potentially eligible will be acquired and subsequently evaluated in detail. Eligibility will be assessed based on the following criteria:

- Population: patients with histologically confirmed adenocarcinoma of the rectum, UICC stage II/III disease, and locally resectable disease
- Intervention: neoadjuvant fluoropyrimidine-based chemoradiotherapy with the addition of a platinum derivative; trials evaluating neoadjuvant chemoradiotherapy with any other combined regimen (e.g., irinotecan, monoclonal antibodies) or neoadjuvant chemoradiotherapy followed by neoadjuvant chemotherapy before surgery will be excluded.
- Comparator: fluoropyrimidine-based single-agent neoadjuvant chemoradiotherapy; trials assessing neoadjuvant radiation without chemotherapy will be excluded.
- Outcomes: trials providing data on at least one of the following outcomes will be included:
- Primary outcome parameters: overall survival and disease-free survival
- Secondary outcome parameters: local recurrence rate, distant recurrence rate, and rate of pathological complete response; toxicity; postoperative morbidity and mortality; rate of anastomotic leakage; treatment compliance; and quality of life (QoL).
- Design of primary studies: RCTs; all non-randomized studies will be excluded.

In case of multiple publications on the same clinical trial, data with the longest follow-up will be used for each outcome. If available, the evidence will be supplemented by unpublished data as a separate subgroup. The study selection process will be documented by a PRISMA flow diagram giving specific reasons for exclusion of studies at each stage.

### Data extraction

Data will be extracted from the trials that meet our final inclusion criteria using a standardized, electronic data extraction form. Two reviewers will independently extract data using the standardized form, and extracted data will be compared between the two extracting reviewers. Any queries/discrepancies will be resolved by consultation of a third party. The following trial characteristics and outcomes will be extracted:

1. Design and reporting features: first author, year of publication, location of trial conduct, journal, publication language, details of trial design (treatment arms, superiority/non-inferiority design, etc.), duration of the trial, number of trial centers (single-center/multicenter), sample size calculation (yes/no/not stated; *α*, *β*, and *δ* in %), follow-up time (in months/not stated), and trial registration
2. Quality features: according to the Cochrane Risk of Bias Tool (see section “Qualitative analysis” below)
3. Patient characteristics: mean age, gender distribution, oncologic performance scale (WHO scale, ECOG scale, Karnofsky index, etc.), UICC stage, TNM stage, and height of the tumor
4. Treatment characteristics: details of chemotherapeutic regimen, dosage and fractions of radiation, details of surgery (low anterior resection; abdominoperineal extirpation; laparoscopic/open), period between the end of radiation and day of surgery (weeks), and details of adjuvant treatment
5. Outcomes: overall survival, disease-free survival, local recurrence rate, distant recurrence rate, rate of pathological complete response, treatment toxicity, postoperative morbidity and mortality, rate of anastomotic leakage, treatment compliance, and QoL

In case of missing information or ambiguities in the publications of individual trials, the trial authors will be contacted to retrieve additional information.

### Endpoints of the systematic review

The main endpoints of the current systematic review will be survival assessed as overall and disease-free survival. The ultimate goal of cancer treatment is to prolong overall survival. However, for colorectal cancer, disease-free survival has been shown to correlate well with overall survival [33]. Furthermore, Glynne-Jones et al. have recently assessed that disease-free survival represents the best outcome parameter for phase III clinical trials in rectal cancer [29]. The patient relevance of this outcome is obviously high.

Secondary endpoints will include rates of local and distant recurrence and pathological complete response rate as surrogate parameters for efficacy of the different strategies. Furthermore, treatment toxicity, postoperative morbidity, and mortality including anastomotic leakage will be assessed for evaluation of safety. Treatment compliance will be evaluated to assess feasibility of the different treatment protocols. Finally, quality of life will be evaluated as a patient-oriented outcome parameter to judge the tolerability of the different therapeutic strategies.

If differing definitions of the endpoints stated above are used in individual trials, this will be reported and potential implications for the results will be discussed.

During the planning phase of the current systematic review, several representatives of patient organizations (German ILCO e.V., various local groups) have been contacted and responded that survival, quality of life, and treatment toxicities would be of utmost importance from a patient’s perspective.

### Qualitative assessment of included trials

Risk of bias in included RCTs will be analyzed using the Cochrane Collaboration’s tool for assessing the risk of bias in randomized trials [34]. Two reviewers will independently assess risk of bias of each trial. Disagreements will be resolved by discussion or by consulting a third reviewer. Each of the following domains will be evaluated at study level: random sequence generation, allocation concealment, blinding of participants and personnel, blinding of outcome assessment, incomplete outcome data, selective outcome reporting, and other bias (e.g., baseline imbalance, surgical experience, early termination of the trial, funding bias, and deficiencies of the statistical analysis) [35]. Each potential source of bias will be graded as high, low, or unclear, and a quote from the study report together with a justification for the judgment will be presented in the risk of bias table.

The quality assessment results will directly be used to grade the body of evidence at outcome level. This will be done using the GRADE system [36]. The following issues will be considered in analogy to current publishing recommendations: study quality/risk of bias, consistency of results between studies, directness of evidence/generalizability, and magnitude of the effect.

### Statistical analysis

For time-to-event outcomes (primary outcomes), the hazard ratio (HR) with its 95% confidence interval will be chosen as an effect measure per trial. If possible, log HRs and their standard errors will be extracted directly, preferably from an adjusted model. If they are not reported but adequate univariate analyses are available, an indirect estimation method will be used [37, 38]. For dichotomous outcomes (all but one secondary outcome), the odds ratio (OR) with its 95% confidence interval will be used as the effect measure per trial; for the ordinal outcome QoL, the (standardized) mean difference ((S)MD) with its 95% confidence interval will be used. HRs and (S)MDs will be combined using the generic inverse-variance method in a meta-analysis, and ORs will be pooled using the Peto method. Random-effects models will be used to calculate overall effect estimates and confidence intervals because we assume the heterogeneity between the *true* effects of the included trials. Nevertheless, results of fixed-effect models will be contrasted with those of the random-effects models and discussed. All results will be investigated for statistical heterogeneity by *I*^2^ statistics. If there is considerable heterogeneity (*I*^2^ > 75%) for an outcome, no meta-analysis will be done [32]. If possible, the following subgroup analyses will be done for the main endpoints to further investigate potential heterogeneity: stage II vs. stage III rectal cancer and adjuvant vs. no adjuvant treatment. Statistical investigation of a potential publication bias based on a test of funnel plot asymmetry will be done if there is a sufficient amount of RCTs (> 10) available for analysis. Nevertheless, sensitivity analyses will be performed with respect to study quality as well as for different dosages and application methods (e.g., 5-fluorouracil vs. capecitabine). Further sensitivity analyses may be conducted during the course of the review, if individual peculiarities of the included trials become apparent. A power calculation according to Jackson and Turner will be performed [39]. All results will be reported following the PRISMA guidelines [40]. Tables for the summary of findings will be set up according to the Cochrane recommendations. Forest plots will present meta-analysis results graphically. R version 3.2.2 or higher [41] and the R meta package version 4.3-2 or higher (developed by Guido Schwarzer) will be used for all statistical analyses.

## Discussion

The proposed systematic review and meta-analysis will condense the highest level of evidence on the strategy of adding a platinum derivative to 5-fluorouracil (5-FU)-based chemoradiotherapy. In order to maximize the quality of the pooled results, only RCTs will be included, since they represent the study type with the lowest risk of bias. Furthermore, strict quality assessment according to the Cochrane Risk of Bias Tool [34] will be performed and the resulting evidence and recommendations will be judged according to the GRADE recommendations [36]. This will allow for reliable recommendations for clinical practice and future research.

Instead of assessing a specific chemotherapeutic protocol, the two strategies will be assessed in a pragmatic approach considering all types of single-agent 5-FU-based chemoradiotherapy to all combined protocols including a platinum derivative. The wide patient selection criteria of all patients with locally advanced rectal cancer (UICC stage II or III disease) will allow for better generalizability and representativeness of the results.

Apart from the main survival endpoints, treatment toxicity and operative morbidity will be evaluated to judge the safety of the different treatment strategies. Treatment toxicity of intensified chemotherapeutic protocols is of high patient relevance and is of utmost importance for the assessment of comprehensive applicability of such protocols. Furthermore, it has been shown that neoadjuvant treatment has a direct influence on postoperative morbidity and complications such as anastomotic leakage [42, 43]. Therefore, these endpoints have to be evaluated in an attempt to appraise the different treatment concepts comprehensively. On the basis of the results for the efficacy endpoints (survival) on the one hand and the safety endpoints (toxicity/postoperative morbidity) on the other hand, a risk/benefit assessment of the different treatment strategies will be performed. Finally, QoL will be assessed as a key patient-oriented outcome in addition to the abovementioned endpoints to judge the two strategies by a patient’s perspective.

The findings of this systematic review and meta-analysis will thus provide a broad picture of the clinical topic and may form a basis for future clinical decision-making, guideline evaluation, and research prioritization.

## Additional file

Additional file 1: PRISMA-P checklist. (DOC 82 kb)

## Abbreviations

(S)MD: (Standardized) mean difference
5-FU: 5-Fluorouracil
ECOG: Eastern Cooperative Oncology Group
HR: Hazard ratio
MeSH: Medical Subject Headings
OR: Odds ratio
PICO: Participants, interventions, comparisons, and outcomes
QoL: Quality of life
RCT: Randomized controlled trial
UICC: Union for International Cancer Control
WHO: World Health Organization
